# Endogenous Control Mechanisms of FAK and PYK2 and Their Relevance to Cancer Development

**DOI:** 10.3390/cancers10060196

**Published:** 2018-06-11

**Authors:** Rayan Naser, Abdullah Aldehaiman, Escarlet Díaz-Galicia, Stefan T. Arold

**Affiliations:** King Abdullah University of Science and Technology (KAUST), Computational Bioscience Research Center (CBRC), Division of Biological and Environmental Sciences and Engineering (BESE), Thuwal 23955-6900, Saudi Arabia; rayan.naser@kaust.edu.sa (R.N.); Abdullah.aldehaiman@kaust.edu.sa (A.A.); miriam.diazgalicia@kaust.edu.sa (E.D.-G.)

**Keywords:** dimerization, miRNA, motility, anoikis, chaperon, PTEN, FIP200, LKB1, PI3K, regulation

## Abstract

Focal adhesion kinase (FAK) and its close paralogue, proline-rich tyrosine kinase 2 (PYK2), are key regulators of aggressive spreading and metastasis of cancer cells. While targeted small-molecule inhibitors of FAK and PYK2 have been found to have promising antitumor activity, their clinical long-term efficacy may be undermined by the strong capacity of cancer cells to evade anti-kinase drugs. In healthy cells, the expression and/or function of FAK and PYK2 is tightly controlled via modulation of gene expression, competing alternatively spliced forms, non-coding RNAs, and proteins that directly or indirectly affect kinase activation or protein stability. The molecular factors involved in this control are frequently deregulated in cancer cells. Here, we review the endogenous mechanisms controlling FAK and PYK2, and with particular focus on how these mechanisms could inspire or improve anticancer therapies.

## 1. Introduction

Focal Adhesion Kinase (FAK) is a multi-domain non-receptor protein tyrosine kinase (PTK) found in metazoans and also in the unicellular eukaryote *Capsaspora owczarzaki* [1,2]. FAK is a multifunctional protein that integrates and transduces signals perceived through integrin or growth-factor receptors into cytoplasmic and nuclear responses. This mechanism allows FAK to link the functions of adhesion, migration and survival in cells [3,4]. In particular, FAK is an essential factor in embryogenesis and wound healing due to its ability to override apoptosis following cell detachment (anoikis). While present in low levels in most adult tissues, FAK is overexpressed in most types of cancer in which its capacity to override anoikis and to drive cell motility promotes aggressive invasiveness and metastasis of cancer cells [5,6,7,8]. The observation that overexpression of FAK is linked to tumor invasiveness has made FAK a promising target for small-molecule inhibitors [7,9]. Although targeting FAK alone had only limited success in a clinical setting, initial proof-of-principle studies have highlighted that inhibition of FAK not only reduces tumor spreading, but it also makes cancer cells more susceptible to chemotherapy [10,11]. Indeed, FAK activity appears to trigger survival mechanisms under various stress conditions, such as DNA damage or immunological stress [10]. Consequently, promising preclinical results were obtained when FAK inhibitors were administered in combination with BRAF inhibitors or used jointly with chemotherapy, radiotherapy [12] or immunotherapy [13,14]. These combined approaches are now being tested in the clinical settings and are reviving interest in FAK inhibitors that waned due to the limited efficacy of targeting FAK in single-agent therapies.

Improved or novel FAK inhibitors may have a positive impact in combined approaches, because most available FAK inhibitors lack specificity and/or efficacy, and their precise molecular mechanism remains in many cases poorly defined [10]. Moreover, proline-rich tyrosine kinase 2 (PYK2), a close paralogue to FAK in vertebrates [15], can often functionally compensate for loss of FAK, and may thus override therapeutic inhibition of FAK in tumors [16,17]. Although similar in structure and function, FAK and PYK2 differ in their tissue distributions. Whereas FAK is widely expressed, PYK2 is expressed mostly in the brain, osteoclasts, macrophages and lymphocytes [18]. Despite their functional redundancy in many settings, FAK and PYK2 can also play different or even opposing cellular roles. For example, activation of FAK promotes cell cycle progression and survival, whereas activation of PYK2 generally inhibits such events [5,19,20,21,22,23,24]. Hence, under some circumstances (tissue-) specific inhibition of FAK or PYK2 might help an inhibitor to achieve desired therapeutic effects.

In this review, we summarize current understanding of the endogenous mechanisms used by cells to contain aberrant FAK and PYK2 activity. Thorough understanding of these mechanisms might lead to the design of new or improved therapeutic interventions that could block aggressive spreading of cancer cells. The multitude of cellular functions and locations of FAK and PYK2 have been comprehensively described in recent reviews [25,26,27,28]. We thus only briefly outline the general molecular mechanism for FAK and PYK2 activation. We particularly focus our review on how this mechanism is controlled by cellular factors, including alternatively spliced forms, interacting proteins and non-coding RNAs. We also discuss if and how the current knowledge of endogenous control mechanisms of FAK and/or PYK2 could improve the development anti-cancer strategies.

### Mechanistic Bases for Activation of FAK and PYK2

**Figure 1:**
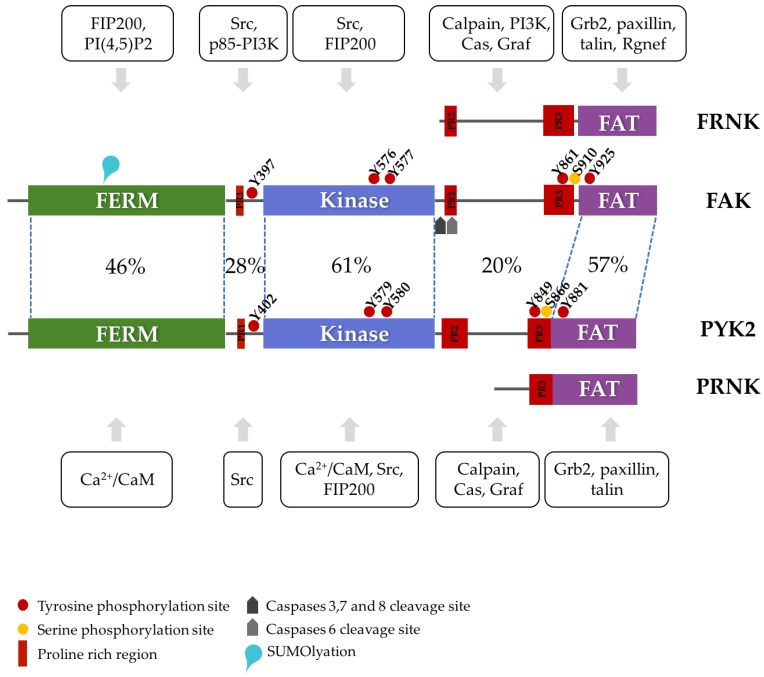
Schematic domain structure of focal adhesion kinase (FAK) and PYK2. The three folded domains are shown in green, blue and magenta. Interaction motifs, sites of post-translational modification, and examples of binding sites of proteins discussed in the text are shown. The percentages show the sequence identity between corresponding regions of FAK and PYK2. The alternatively transcribed products FRNK and PRNK are schematically represented with respect to FAK and PYK2. Interacting proteins for FAK or PYK2 are shown boxed either above (FAK) or below (PYK2) the schematic structure with arrows pointing at the interacting domain or linker region. For a more complete list of interacting partners, please see [26].

PYK2 evolved from FAK through gene duplication and subsequent specialization in vertebrates [15]. In humans, both proteins share 46% sequence identity (65% similarity) and have the same three-domain organization [25,28,29]: an N-terminal band 4.1, ezrin, radixin, moesin (FERM) domain, a central catalytic kinase domain and a C-terminal focal adhesion targeting (FAT) domain (reviewed in detail by [26,28,30]. The domains are connected by long linkers that also contain important protein binding sites (Figure 1) [28,31,32]. Owing to the common origin and high sequence similarity of FAK and PYK2, the basic mechanisms for the (in)activation of one likely remain conserved in the other.

Both FAK and PYK2 harbor binding sites for many proteins (more than 50 have been described for FAK [26]) and nuclear localization and export signals [33,34]. These interaction sites allow FAK and PYK2 to function as molecular scaffolds. For a subset of their functions, the scaffolding function is sufficient, and kinase activity is not required [35,36]. In FAK, the FERM domain can directly bind to the kinase domain and inhibit its function [31]. Key residues for the FERM:kinase interaction are conserved between FAK and PYK2 (in particular FAK F596/PYK2 F599; [26]), suggesting that this FERM:kinase association also exists in PYK2. Experimental evidence for this association in PYK2 is, however, lacking [37,38,39]. In both proteins, kinase activity is generally initiated by autophosphorylation of a tyrosine situated in the FERM-kinase linker (FAK Y397 and PYK2 Y402). Together with the proline-rich region 1 (PR1) situated upstream in the same linker, the phosphotyrosine (pY) forms a high-affinity binding site that recruits and activates Src kinases via a dual interaction with their SH2 and SH3 domains [28]. The bound and activated Src kinase contributes substantially to the kinase activity associated with FAK or PYK2. Under most conditions, autophosphorylation can only proceed in trans, and it requires FAK/PYK2 dimerization and/or clustering [40,41,42,43,44,45]. However, in neurons, alternative splicing results in forms of FAK capable of autophosphorylation in cis, suggesting different regulatory modes in the brain [43].

**Figure 2:**
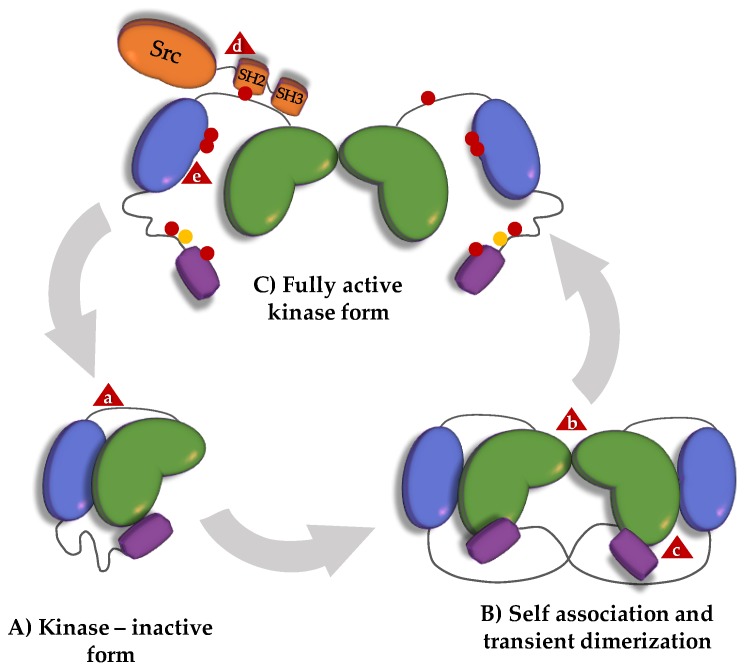
Canonical FAK activation scheme. (**A**) In the absence of integrin activation, an interaction between the FERM and kinase domain (indicated by a lower-case ‘a’ in a red triangle) inhibits FAK kinase activity. (**B**) Ligand-mediated recruitment and clustering of FAK at focal adhesions promotes transient dimerization by stabilizing weak FERM:FERM interactions (lower-case ‘b’ in a red triangle) and promoting FERM:FAT binding in trans (lower-case ‘c’ in a red triangle). (**C**) FAK clustering and self-association allows trans-autophosphorylation of Y397 (red dots) in the FERM-kinase linker. When phosphorylated, Y397 and PR1 form a bidentate binding site for the SH2 and SH3 domains of Src (lower-case ‘d’ in a red triangle). Recruitment-activated Src phosphorylates the activation loop of the FAK kinase domain (lower-case ‘e’ in a red triangle) and other tyrosines on FAK, resulting in an open FAK conformation and full enzymatic activity. Triggered signaling may result in additional FAK modifications (e.g., serine phosphorylation; yellow dots) and may ultimately lead to dephosphorylation and/or displacement of FAK from focal adhesions (back to the closed monomeric inactive conformation of A), or to proteolytic cleavage and degradation (not shown in figure).

In FAK, dimers are formed through FERM:FERM and FERM:FAT interactions (Figure 2) [40]. In PYK2, FERM domains form crystallographic dimers that are identical to those formed by the FAK FERM domains (PDB 4eku), suggesting that the FERM:FERM dimerization mechanism might be conserved. Self-association of FAK and PYK2 is controlled by ligands. In FAK, trans-autophosphorylation, and thus kinase-dependent functions occur mostly at focal adhesions to which FAK is recruited, enriched and primed for dimerization via interactions between its FAT domain and the leucine-aspartic acid (LD) motifs of paxillin [40]. Additionally, clustering of FAK is promoted through interactions between the FERM domain and membrane-associated phospholipids [46]. Although the FAT domain in PYK2 can bind to paxillin [47], PYK2 is not as strongly localized as FAK is to focal adhesions in most cell types. Conceptually similar recruitment/clustering mechanisms appear to lead to activation of FAK and PYK2 at other transmembrane receptor complexes, such as T-cell immune synapses [48,49], or to activation of PYK2 in post-synaptic densities [50]. In addition, FAK and PYK2 play roles in various other cellular locations, including endosomes, adherens junctions, the microtubule organizing center and the nucleus [3,25]. Some of these functions (e.g., at nascent adhesions or in the nucleus) are kinase-independent.

A major difference between FAK and PYK2 is that the latter can be activated by calcium ions (Ca^2+^). The exact molecular mechanism for activation of PYK2 by Ca^2+^ remains unclear and controversial. The current view is that Ca^2+^ does not bind directly to PYK2, but binds through calmodulin (which has been reported to bind to either the FERM [45] or kinase domain of PYK2 [51] or through Ca^2+^-activated PYK2-modifying kinases (PKC, CaMKII, PKA), phosphatases (calcineurin, PP1) or Ca^2+^-activated proteins (PSD-95) [26,52].

In summary, FAK and PYK2 are versatile protein scaffolds with kinase-dependent and kinase-independent functions. Although the details may differ, the underlying mechanism for the control and activation of kinase activity appears to be preserved between FAK and PYK2. Activation of the kinase function requires autophosphorylation. Cellular ligands can trigger trans-autophosphorylation by stabilizing a weak intrinsic propensity for self-association of FAK or PYK2 and/or by increasing the local protein concentration. Consequently, endogenous inhibitory mechanisms can prevent kinase activation by lowering FAK and/or PYK2 protein concentrations, by reinforcing inactivating autoinhibitory interactions, by competing with activating ligands, by dephosphorylating the Src-recruiting tyrosine or by displacing FAK/PYK2 from their sites of activation (Figure 1). 

## 2. FRNK and PRNK

Both the FAK and PYK2 genes (PTK2 and PTK2B, respectively) can produce shorter protein forms that lack the FERM and kinase domains [53,54]. These autonomously expressed non-cataytic C-terminal fragments presumably originate from alternative transcription initiation sites [55] and are called the FAK-related non-kinase (FRNK) and the PYK2-related non-kinase (PRNK). Starting at residues 693 and 780 of the canonical FAK and PYK2 isoforms, respectively, they include protein binding sites (proline-rich sites PR2 and PR3 for FRNK and PR3 for PRNK), phosphorylation sites (most prominently Y861 and S910 in FAK and Y849 and S866 in PYK2) and the FAT domain (Figure 1). PR2 and PR3 mediate the binding of different SH3-containing proteins, such as p130cas or Graf [54,56,57,58,59,60]. The FAT domain is sufficient to localize to focal adhesions, via interactions mainly with paxillin, but also with other protein ligands, such as talin or Rgnef/p190RhoGef [61,62,63]. Alternative interactions can also localize FAT to other structures, such as the T-cell receptor complex [48,64], or the growth cone of developing axons [65]. FRNK and PRNK maintain these functions, although without any regulatory influence from the other FAK/PYK2 regions. The production of these truncated forms has therefore been suggested to lead to a direct competition between FRNK or PRNK and FAK and/or PYK2 at sites that attract their FAT domains. Indeed, immunostaining showed that FRNK and PRNK localize to focal adhesions [53,54]. By competitively displacing FAK or PYK2, the alternative spliced variants lower the local concentrations of FAK and PYK2 and hence lower the propensity for trans-autophosphorylation of FAK and, possibly, PYK2. Accordingly, western-blot analysis indicates that FAK Y397 phosphorylation, the key step in FAK activation, decreases at increasing FRNK levels [66,67]. Moreover, by displacing FAK or PYK2 and by forming incomplete signaling complexes, FRNK and PRNK promote the disassembly of focal adhesions. Indeed, the expression of FRNK and PRNK correlates with focal adhesion turnover [53,54,68].

In addition to displacing FAK from common binding sites, FRNK may also act by directly binding to FAK. In rat aortic smooth muscle cells, FRNK co-immunoprecipitates with FAK [67]. This association is decreased by phosphorylation of FRNK S217 by the extracellular signal-regulated kinase (ERK1/2, henceforth referred to as simply ERK, unless stated otherwise) and increased by a factor of five by the S217A mutation. In cells, autophosphorylation of FAK was reduced by expression of FRNK, and even more so by expression of the S217A mutant form of FRNK [67]. The molecular basis for this phosphorylation-dependent interaction is unknown, but it might involve competition of FRNK with the intramolecular FERM:FAT interaction that promotes FAK for autophosphorylation by reinforcing FAK dimers. In size exclusion chromatography, dimerization of recombinant FAK was indeed weakened by the presence of recombinant FAT domains [40]. FRNK S217 corresponds to Erk-phosphorylated S910 in the canonical FAK isoform. This serine is situated five residues upstream of the four-helix FAT domain. The S910PPP motif weakly interacts with the FAT domain [69] and, when phosphorylated on S910, constitutes a proline-isomerase binding site [67,70]. Phopshorylation of FRNK S217 could therefore affect the FRNK:FAK association in various direct or indirect ways.

The possibility that a similar mechanism of competitive binding also exists for PRNK and PYK2 has not been tested. If this mechanism does exist, then this association would be regulated differently, because the PYK2 serine 866, which is also phosphorylated and located on the N-terminal side of its FAT domain, is not situated in an ERK phosphorylation motif [71].

## 3. Proteases

FAK and PYK2 are cleaved by several proteases. Some of the resulting fragments resemble FRNK or PRNK.

### 3.1. Caspases

Cysteine aspartate-specific proteases (caspases) play an important role in programed cell death (apoptosis). During apoptosis, caspases cleave critical repair and structural proteins that bolster cell survival. It has been shown that FAK is cleaved in the early stages of myc-induced apoptosis [72,73,74]. Sensitivity profiling of protease inhibitors showed that FAK is cleaved by caspase-3, 6, 7 and 8, with caspase-7 by far the most active [75,76]. Caspase-3, 7 and 8 generated 85 kDa and 33 kDa fragments [75,76]. Caspase-6 cleaved FAK with low efficiency, generating a 77 kDa fragment [76]. Generated cleavage products and cleavage site consensus suggested that the 85-kDa cleavage happened after D772 and that the 77 kDa cleavage occurred after D704 (Figure 1) [76]. Accordingly, the D772A mutation was not cleavable by caspase-3 [76]. It is expected that the separation from the FAT domain weakens the attachment of the FERM-kinase domain fragment to focal adhesions (and other FAT-targeted sites), leading to turnover of these structures. The 35 kDa fragment generated from FAK cleavage resembles FRNK and may act similarly. Indeed, western-blot analysis indicated a decrease in FAK Y397 phosphorylation levels in HeLa cells transfected with the 35 kDa fragment [76]. No direct evidence for caspase cleavage of PYK2 currently exists.

### 3.2. Calpain

Calpain, a calcium-dependent cysteine protease, cleaves both FAK and PYK2 [77,78]. When either purified calpain I or calpain II was added to FAK, four fragments were generated with sizes of 90 kDa, 50 kDa, 40 kDa, and 35 kDa, and the cleavage was blocked by the calpain inhibitor, calpastatin [78]. Antibodies against the FAK kinase domain reacted with the 90-kDa and 50-kDa bands while antibodies against the C-terminus of FAK bound to the 35 kDa band [78]. PYK2 cleavage products that were 80 kDa and 75 kDa in size were observed in human platelets; when treated with the calpain inhibitor calpeptin, the bands were no longer present [79].

## 4. Regulation through Post-Translational Modifications

In addition to irreversible protease cleavage, FAK and PYK2 are subjected to several types of post-translational modifications. FAK, but not PYK2, has been reported to be acetylated on residue A2 [71] and SUMOylated on K152 in the FERM domain [80]. SUMOylation was mediated by the SUMO ligase protein inhibitor of activated STAT1 (PIAS1) that interacted with FAK in cells and in vitro. SUMOylation was increased in suspended cells and correlated with increased nuclear presence of FAK and increased FAK Y397 phosphorylation independently of cell adhesion [80]. The mechanistic bases for this effect remain to be determined, but since K152 is in the vicinity of the FERM:kinase interaction site [31], its SUMOylation might interfere with the ‘closed’ autoinhibitory FAK conformation.

The most important post-translational modification is (de)phosphorylation [81]. According to the UniProt database, FAK and PYK2 are phosphorylated on serines (8 on FAK; 6 on PYK2), threonines (FAK: 2; PYK2: 2) and tyrosines (FAK: 8; PYK2: 8). Regulation through phosphorylation was previously reviewed in detail [4,28,71,82]. Here, we present a very broad overview. Tyrosine phosphorylation is generally associated with activating characteristics, starting of course with the autophosphorylation of the FERM-kinase linker tyrosine (Y397 and Y402 in FAK and PYK2, respectively). Src-mediated phosphorylation of a tyrosine in the first FAT helix (FAK-Y925 and PYK2-Y881) triggers activation of mitogen-activated protein (MAP) kinases via the phosphotyrosine-binding adapter protein Grb2 [83,84,85,86]. In FAK, phosphorylation of S910 by ERK recruits the proline-isomerase PIN1 and subsequently the protein tyrosine phosphatase (PTP-PEST), which then counteracts FAK phosphorylation and activation, thus closing a negative feedback loop [70,87]. In PYK2, a nuclear export signal in its kinase-FAT linker is activated by phosphorylation of S778 by protein kinase A (PKA) and deactivated by the phosphatase calcineurin [34]. Conversely, PKC-mediated phosphorylation of FAK on S722 promoted nuclear localization [88]. (De)phosphorylation therefore regulates nuclear localization of PYK2 and FAK, although via different mechanisms. Phosphorylation can also alter the protein structure, as seen in FAK activation loop tyrosines. Their phosphorylation not only increases the kinase catalytic activity by restructuring the activation loop, but it also prevents the kinase domain from forming an inhibitory interaction with the FERM domain [31].

While most kinases or phosphatases probably form only transient or short-lived complexes with FAK and/or PYK2, the tyrosine-kinases Src and Fyn durably attach to the Y397-phosphorylated FERM-kinase linker. The resulting protein complexes are associated with a catalytically active state [4,28,89,90,91]. In the following, we highlight one other kinase and one phosphatase that also have lasting associations with FAK or PYK2 and exhibit inhibitory effects.

### 4.1. Liver Kinase β1 (LKB1)

**Figure 3:**
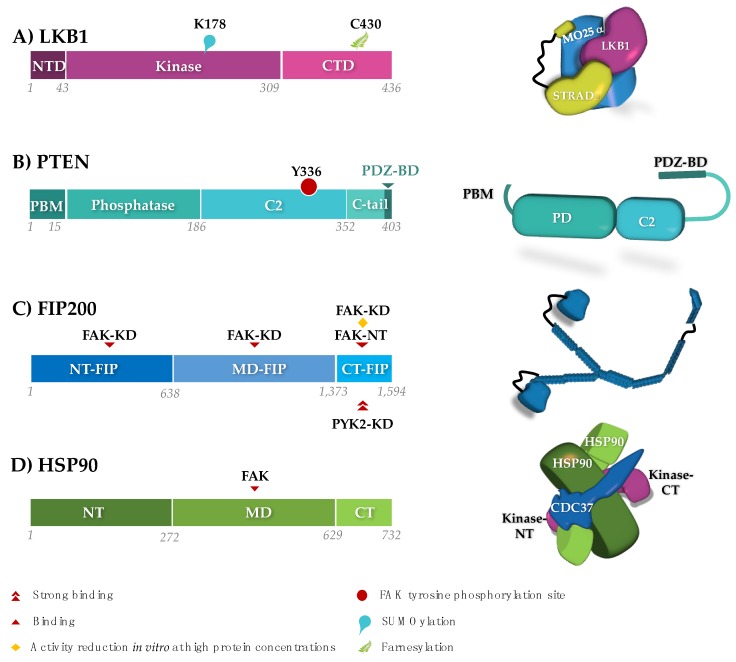
Schematic domain structures (left) and three-dimensional (3D) architectures (right) of protein regulators of FAK:LKB1, PTEN and FIP200 are negative regulators. LKB1 is shown as part of the activating complex formed with MO25α and STRADα (according to PDB accession 2wtk). The presented 3D structure of FIP200 (blue) is highly speculative and based on secondary structure predictions and homology modeling. The stabilizing function of HSP90 is upregulated in cancers. HSP90 is displayed with the kinase-specific adaptor CDC37, maintaining the separated N-terminal (NT) and C-terminal (CT) lobe of a kinase domain (taken from PDB 5fwl). Binding sites and post-transcriptional modifications relevant to their interaction with FAK and PYK2 are indicated. Abbreviations are: (**A**) LKB1:NTD, N-terminal domain; CTD, C-terminal domain. (**B**) PTEN:PD, phosphatase domain; PBM, PI(4,5)P2-binding module; PDZ-BD: PDZ binding domain. (**C**/**D**) FIP200/HSP90:NT, N-terminal domain; MD: middle domain; CT, C-terminal domain. FAK:KD, kinase domain; NT, N-terminal fragment.

The liver kinase β1 (LKB1) or STK11 is a multifunctional serine/threonine kinase that links energy sensing and cell polarity [92,93,94]. In particular, LKB1 stabilizes cell polarity under energy stress. Given that loss of cell polarity increases undirectional ‘exploratory’ migration, LKB1 controls cell movements during embryogenesis and wound healing [92,93,94]. LKB1 also counteracts cancer cell metastasis and is the third most frequently mutated gene in lung adenocarcinoma [95,96,97]. Rather than through phosphorylation of its activation loop, LKB1 (Figure 3) is activated allosterically through an association with the pseudo-kinase STRADα, which is tethered to LKB1 by the scaffolding protein MO25α [98]. In addition, LKB1 activity and localization are affected by post-translational modification [92,94]. SUMOlyation of LKB1 K178, in response to energy stress, promotes the interaction between LKB1 and the AMP-activated protein kinase (AMPK). The resulting phosphorylation and activation of AMPK help to maintain the energy balance in a cell during energy stress [99]. Furthermore, live cell imaging experiments show that farnesylation on C430 within the LKB1 C-terminus is required for its co-localization with actin at the leading edges of migrating cells. The LKB1 kinase activity is required to stabilize the actin colocalization of LKB1 and to promote mesenchymal polarization and directed cell migration [93,94].

Initially, LKB1 was linked to FAK through the observation that the FAK adhesion pathway is upregulated in LKB1^−/−^ mice in a mutant Kirsten rat sarcoma (KRAS) background [100]. Subsequent analyses confirmed that the presence and activation of LKB1 represses phosphorylation of FAK Y397, Y861 and Y925 [93,101]. The current model proposes that farnesylation colocalizes LKB1 with actin at the leading cell edge of migratory cells, where the LKB1 kinase activity indirectly decreases FAK activation. Inactivation of FAK decreases focal-adhesion turnover and hence stabilizes focal adhesions at the leading edge. Conversely, loss of LKB1 farnesylation or kinase activity results in FAK hyperphosphorylation and leads to cells that present exploratory behaviors with loss of directional persistance [92,93].

The exact mechanisms by which LKB1 negatively affects FAK activation are unclear. FAK and LKB1 can co-immunoprecipitate each other, suggesting that they colocalize within the same protein complex. However, purified FAK and LKB1/MO25α/STRADα proteins did not interact in vitro, suggesting that the interaction between FAK and LKB1 may not be direct [93]. One possibility would be that the effect of LKB1 is mediated by its downstream substrates. Activation of AMPK or its family members (e.g., MARK1 and MARK4) correlates with inhibition of FAK phosphorylation in several systems, including human liver cancer cell lines and muscle cells [101,102,103,104]. The details of these inhibitory pathways through AMPK or MARKs remain incompletely established. Intriguingly, one study could recapitulate the FAK pY397-suppressing effect of LKB1 by expressing just the LKB1 N-terminal arm region (residues 1–47) in LKB1-null H157 cells, suggesting that, at least in this setting, LKB1 kinase activity was not needed [93]. The possibility of an interaction between PYK2 and LKB1 has not yet been explored, although PYK2 activation has been observed following AMPK activation in muscle cells [105,106].

### 4.2. PTEN

The phosphatase and tensin homolog deleted on chromosome 10 (PTEN) (Figure 3) exerts a dual function as both a lipid and a protein phosphatase [107,108]. As a lipid phosphatase, PTEN converts phosphoinositide(3,4,5)P3 to phosphoinositide(4,5)P2, and is therefore the antagonist of the phosphoinositide-3 kinase (PI3K). PI3K directly associates with FAK through its PR motifs [109] promoting cell survival via the activation of the PI3K/Akt pathway. This association has an important effect on maintaining cell adhesion to integrins, which is, in some tissues like endothelial cells, indispensable for cell survival [110].

PTEN affects cell migration and invasiveness by dampening this FAK/PI3K pathway. The most commonly observed effect of PTEN on FAK might be indirect through its lipid-phosphatase-based downregulation of the PI3K pathway [111]. As an additional indirect mechanism, PTEN was shown to inhibit FAK expression levels in myeloma and gastric cancers [112,113]. However, PTEN can also form a complex with Y397-phosphorylated FAK in glioma, breast cancer and colon carcinoma cells [114,115]. This interaction leads to dephosphoryation of pY397 following cell detachment in these cells. The FAK:PTEN association was compatible with the FAK:paxillin association, but it competed with binding of PI3K and Src to FAK [114,115]. Both the p85 subunit of the PI3K and the SH2-SH3 fragment of Src kinase bind to the Y397-phosphorylated FERM-linker fragment [4,28,89,90,116,117], suggesting that PTEN binding involves the same region. Moreover, it has been observed that FAK phosphorylates PTEN at Y336 with a positive effect on PTEN stability and phosphatase activity [118,119]. Y336 is located in the phospholipid-binding region of the C2 domain (Figure 3). In principle, the introduction of a negative charge in this position is expected to hamper association with negatively charged lipid headgroups. Future research is needed to clarify the underlying molecular mechanisms causing the multiple effects of the FAK:PTEN association.

## 5. Regulation through Non-Catalytic Protein Interactions

FAK or PYK2 also have a large number of interaction partners that do not post-translationally modify them. Many of these partners either control subcellular localization, or attach to FAK or PYK2 to form active signaling complexes [4,28,117]. So far, to our knowledge, only one non-catalytic protein has been identified as an inhibitor of FAK and PYK2 activation, namely FIP200.

### RB1CC1/FIP200

In vitro and in vivo protein interaction assays led to the discovery of a 200 kDa interacting protein for both FAK and PYK2, named the FAK family kinase-interacting protein (FIP200) [120]. The 1594 residue FIP200 is predicted to contain an N-terminal ubiquitin-like domain (ULD) but would otherwise be mostly composed of loops and long helices, possibly forming coiled-coiled structural elements [120,121] (Figure 3). Accordingly, FIP200, also known as the RB1-inducible coiled-coil protein 1 (RB1CC1), is a non-catalytic scaffolding protein. It regulates a wide range of cellular events, such as growth, proliferation, apoptosis and autophagy, through interactions with signaling proteins (e.g., PYK2, FAK, ActA, p53, TSC1, ASK1, TRAF2 and Stathmin) [122]. 

Immunofluorescence analyses of FIP200 indicated a mostly diffused localization in the cytoplasm [120]. But the presence of a consensus nuclear localization signal implies an alternative function for FIP200 in the nucleus, where it may act as a transcription factor [120,121,123,124]. Additionally, FIP200 polyclonal antibody pull-downs revealed a partial colocalization of the protein in focal contacts where it may regulate FAK and vinculin [125]. FIP200 binding decreases the kinase activity of FAK and PYK2 [120,125] and the autophosphorylation levels of FAK [125]. During cell adhesion to fibronectin, FAK activation correlates with increased dissociation of FAK and FIP200 [122,125].

Given that FIP200 inhibits both FAK and PYK2, which can have opposing functions, for example in the promotion of the cell cycle and apoptosis, the outcome of their inhibition by FIP200 may be cell-type specific. For example, overexpression of FIP200 in glioblastoma cells (where PYK2 has dominant functions) leads to inhibition of apoptosis [126], whereas in breast cancer cells, FIP200 acts as a tumor suppressor gene [124].

Although the direct interaction with FIP200 appears to inhibit both FAK and PYK2 in a similar manner, the way in which FIP200 physically binds them might differ (Figure 3). In one study, the FIP C-terminal (CT-FIP; residues 1374–1591) interacts strongly with the PYK2 kinase domain. However, CT-FIP only binds to the N-terminal region of FAK, while the FAK kinase domain (residues 403–672) binds to the FIP200 N-terminus (NT-FIP; residues 1–638) and the middle domain (MD-FIP; residues 639–1373) [125]. Conversely, NT-FIP and MD-FIP did not show affinity for PYK2. The apparent differences might be caused by a change in the relative affinity of the fragments of FIP200 for FAK or PYK2. For example, although CT-FIP did not display significant binding to the FAK kinase, it could reduce its enzymatic activity when present at high concentrations in in vitro kinase assays [125,126]. The differences in binding affinity might have important functional implications in cases where FAK and PYK2 are present, but these functions might be different. For example, in retinoblastoma cells, the interaction of CT-FIP with PYK2 is stronger, and hence preferred, than the interaction of the other two domains of FIP200 with FAK [120,125,126].

## 6. Chaperones

Molecular chaperones can interact with exposed hydrophobic patches of misfolded proteins and help them to (re)establish their native and functional three-dimensional structure. While chaperones commonly release refolded clients, some remain associated with structurally fragile proteins to sustain their function. Thus, chaperones maintain cellular proteins in their functional state and prevent their degradation [127,128]. Overexpression of chaperones, in particular of inducible heat shock proteins (HSPs), is prevalent in cancer cells where proteotoxic stress is endemic [129,130]. The resulting increase in protein stablity and function may enhance the action of overexpressed and/or mutated oncogenes and more generally help to create a protective and resistant cytoplasmic environment [131]. Inversely, a decrease in the expression of molecular chaperones is associated with accumulation of misfolded proteins and the onset of neurodegenerative diseases [131]. 

In a systematic survey of the kinome, FAK and PYK2 were identified as ‘weak clients’ for HSP90 and its kinase-specific cochaperone, CDC37 [128]. Xiong et al. (2014) reported an interaction between HSP90β and FAK in breast cancer cells [132]. FAK bound to the middle domain of HSP90β (residues 233–620), in agreement with a structural analysis of the complex formed by HSP90, CDC37 and a client cyclin-dependent kinase (CDK4) [133]. The HSP90β interaction protects FAK from ubiquitylation-dependent proteasomal degradation. Inhibition of both HSP90β and FAK reduces tumor growth in breast cancer cells [132].

Chaperones can also have more indirect effects on FAK activity. Caino et al. showed that inhibition of mitochondrial HSP90 chaperones, including the tumor necrosis factor receptor-associated protein-1 (TRAP-1), leads to a stress-triggered activation of the LKB1-AMPK pathway [134]. AMPK phosphorylates and activates Unc-51-like autophagy activating kinase (ULK1), which in turn phosphorylates FIP200. These events activate the autophagy-initiating complex between ULK1, FIP200 and autophagy-related protein 13 (atg13), and they allow activated FIP200 to maintain FAK in an inactive unphosphorylated state [134]. Increased expression and increased function of mitochondrial HSP90, as observed in some cancers, lead to sufficient ATP production in nutrient-scarce tumor cells to avoid triggering activation of the LKB1-AMPK pathway. This releases the inhibitory effect of FIP200 on FAK, results in increased cytoskeletal dynamics and enhances invasion of tumor cells into bone or liver in mice disease models [134].

Additionally, heat shock proteins HSP70 and Mamalian relative DNA J (MRJ, also named DNAJB6) exert an oncogenic effect on colon cancer cells. HSP70 and MRJ interact with the urokinase-type plasminogen activator receptor (uPAR) to activate FAK, c-Src, H-Ras, AKT and MAPK cell-signaling pathways, regulating cell adhesion and migration [135]. Another example is the molecular chaperone Cosmc. Cosmc is necessary for the formation of active T-synthase, which catalyses the production of T antigen. Increased expression of T antigen is correlated with tumor metastasis and poor prognosis in colorectal cancer. The forced expression of Cosmc in colon cancer cell lines increases T antigen expression, resulting in increased activation of the FAK, PI3K/Akt and MAPK kinase (MEK)/ERK signaling pathways [136]. Cosmc-induced tumor cell migration and invasion suggest that Cosmc may serve as a potential target for colorectal cancer. The eaxct mechanisms that lead to FAK Y397 phopshorylation through either HSP70/MRJ or Cosmc under these conditions have not yet been determined. Given that FAK activation involves the PI3K and MAPK pathways in both cases, the molecular routes may be overlapping or converging.

## 7. Transcriptional Regulation of FAK and PYK2

The normal or alternative intronic promoters of the *PTK2* and *PTK2B* genes are controlled in a tissue- and developmental-stage—dependent manner [55]. Factors that activate the *PTK2* promoter include the nuclear factor-κB (NF-κB), argonaute 2 (AGO2), PEA3 and NANOG [15,137,138,139]. Overactivity of these *PTK2* transcription factors is associated with tumorigenesis, and their silencing can block tumorigenesis and metastasis [10]. The major reppressor of the human *PTK2* and *PTK2B* promoters is the stress-induced tumor suppressor p53 [140,141,142]. Two putative binding sites for p53 have been indentified in the *PTK2* promoter [143]. In turn, nuclear FAK promotes degradation of p53 by linking it to the E3 ubiquitin ligase Mdm2, using the FERM domain as a scaffold. FERM-triggered proteosomal degradation of p53 leads to cell proliferation and survival under cellular stress [35]. A strong correlation between FAK overexpression and p53 mutations has been observed in human breast cancer [144]. While loss or damage of p53 is closely associated with the development of tumors, forced overexpression can cause premature aging [145]. However, therapeutic restoration of a functional p53 pool at endogenous levels can have tumor-suppressive effects [146].

## 8. Non-Coding RNA

Non-coding RNA (ncRNA) are RNA molecules that are not translated into proteins [147]. ncRNAs include transfer RNA (tRNA), ribosomal RNA (rRNA) and small RNAs. ncRNAs are further classified according to their length and function, and they include long non-coding RNA (lncRNA), micro-RNA (miRNA), small-interfering RNA (siRNA) and piwi-interacting RNA (piRNA). Small RNAs can regulate gene expression at the transcriptional and post-transcriptional level, but they can also affect protein function through direct binding and scaffolding. Many ncRNAs are abnormally expressed in cancer, leading to large-scale deregulation of protein genes, most prominently the tumor suppressor p53 [148]. Here, we focus on those ncRNAs that directly alter expression and function of FAK or PYK2; we note that many other ncRNAs have pleiotropic effects that may also affect FAK or PYK2.

### 8.1. microRNA

miRNAs contain about 20 nucleotides and are transcribed from miRNA genes or coding gene introns. miRNAs post-transcriptionally downregulate target genes by either destabilizing and degrading their mRNA or by binding to the 3′-untranslated region (3′UTR) of their mRNA, thus inhibiting translation [149,150]. miRNAs are implicated in various cellular processes, such as development, cell proliferation, and differentiation. They also regulate junction protein gene expression and extracellular matrix—associated processes, thus helping to maintain the integrity of the cellular structure [151,152]. By inhibiting gene translation, many miRNAs act as tumor suppressors and their downregulation in cancer cells generally promotes cancer formation and metastasis [153]. Conversely, forced upregulation of miRNA can have tumor-suppressive effects, making miRNAs interesting therapeutic targets and prognostic markers for anti-cancer therapy.

#### 8.1.1. Tumor-Suppressor miRNAs Acting Directly on FAK

miR-7. One of the most studied microRNAs regulating FAK is miRNA-7 (miR-7). miR-7 is encoded by three DNA loci (9q21, 15q26 and 19q13) that produce the same mature 23-nucleotide miR-7 sequence. miR-7 is enriched in various normal tissues and implicated in organ development and other biological processes (reviewed by [154]). miR-7 can directly bind to 3′UTR of FAK mRNA, repressing FAK protein expression [155]. miR-7 is also involved in the growth, migration, and invasion of many cancer types. miR-7 downregulates glioblastoma and colon cancer cell invasiveness by repressing FAK protein expression [155,156]. Forced expression of miR-7 significantly reduces endogenous FAK protein expression in breast cancer cell lines, inhibits primary breast tumor growth and invasiveness, and represses metastatic migration of breast cancer xenografts [157]. In non-small-cell lung carcinoma (NSCLC) cells, miR-7 suppresses cell proliferation, migration and invasion by downregulating FAK expression and inhibiting the activation of the ERK/MAPK signaling pathway [158]. The inverse relation between miR-7 expression and tumor growth and invasiveness suggests that miR-7 can be used as a diagnostic marker for certain cancers [154]. miR-7 also positively affects radiation therapy outcomes as it increases cancer cell radio-sensitivity [159], phenocopying an effect observed for FAK-targeting inhibitors [160,161,162]. 

miR-1298. miR-1298 has been identified in a global miRNA functional screen as selectively lethal to cells expressing mutated KRAS, which is an oncogene mutated in 20% of human cancers, mainly NSCLC and colorectal cancers. miR-1298 acts a tumor suppressor in KRAS-driven NSCLC and colorectal cancer cells by directly targeting the mRNA of both FAK and the Laminin subunit beta 3 (LAMB3), lowering their protein expression levels [163]. Interestingly, expression of only LAMB3, and not FAK, is upregulated by KRAS; yet silencing of either FAK or LAMB3 recapitulates miR-1298-induced cell death in KRAS-dependent cancer cells. However, FAK Y925 phosphorylation also links FAK activation via Grb2 to activation of the RAS pathway [164], and FAK signaling is a requirement for the maintenance of KRAS-dependent adenocarcinomas [165].

miR-543. miR-543 expression is decreased in tumorous endometrium tissue and associated with impaired cancer cell invasion in vitro. miR-543 binds to the 3′UTR of FAK and of the TWIST1 oncogene, decreasing their expression at both the mRNA and protein levels. This repression results in impaired tumor cell proliferation, migration and invasion in cancer cell lines [166]. 

miR-379-5p. Underexpression of miR-379-5p in hepatocellular carcinoma (HCC) is correlated with the tumor node-metastasis (TNM) stage and metastasis. miR-397-5p inhibits tumor migration and invasion in HCC cells by direct binding to the 3′UTR of FAK, inhibiting FAK translation while the mRNA level is not affected. This inhibition suppresses AKT activation and hence inhibits the PI3K/AKT pathway. Introduction of miR-397-5p represses HCC metastasis and EMT in vivo [167].

miR-193b. A recent study showed that miR-193b is downregulated in liposarcoma while its reintroduction induces apoptosis in liposarcoma cells. miR-193b functions by directly targeting FAK mRNA and regulating the oncogenic FAK/SRC/BCAR1 signaling pathway [168].

#### 8.1.2. miRNAs Indirectly Regulating FAK

Rather than directly suppressing FAK expression by binding to FAK mRNA, miRNAs can indirectly affect FAK targeting by activating upstream effectors. For instance, miR-205 and miR-940 negatively regulate Src kinase expression. miR-205 is suppressed in renal and prostate cancer. Its inhibition increases cell adhesion in normal keratinocytes, whereas its overexpression reduces tumor growth in vivo [169,170,171]. miR-205 binds to Src 3′UTR, degrading its mRNA, decreasing Src protein expression and hence activating FAK and paxillin [169,170,171]. The exosomal miR-940 also inhibits the expression of Src at the mRNA and protein levels in ovarian cancer, thus decreasing the activation of downstream proteins such as FAK, paxillin and Akt [172].

#### 8.1.3. Oncogenic miRNAs Acting on FAK

Although most FAK-targeting miRNAs act as tumor suppressors by lowering FAK expression and/or activation, some miRNAs show oncogenic effects by targeting cellular inhibitors of FAK [173,174]. The miR-130 family (miR-130b, miR-301a and miR-301b) is one example that is upregulated in bladder cancer tissues. The miR-130 family activates FAK and the Akt signaling pathway by targeting PTEN either directly through binding to its mRNA (miR-130b) or indirectly by repressing PTEN expression (miR-301a/b). High expression of miR-130 increases migration and invasion of bladder cancer cells [173] and the progression of malignant melanoma [174].

Several other miRNAs have been found to influence the activation of FAK by repressing or activating an upstream effector of FAK. Due to space limitations, these miRNAs are listed in Table 1 but not discussed further.

#### 8.1.4. miRNA Regulators of PYK2

Not many miRNAs are known to regulate the expression of PYK2. However, PYK2 is a direct target of miRNA-23b, which binds to its 515-522 3′UTR. Overexpression of miR-23b decreases the protein level of PYK2, but not of FAK, and results in reduced cell proliferation, migration and invasion of hepatocellular carcinoma and glioblastoma cells. Additionally, elevated expression of miR-517a and miR-517c result in decreased PYK2 expression, suggesting that these miRNAs play a tumor-suppressor role in hepatocellular carcinoma [211]. 

### 8.2. LncRNAs

NcRNAs with more than 200 nucleotides, commonly called lncRNAs, are implicated in many biological and pathological processes [212]. The oncogenic lncRNA metastasis-associated lung adenocarcinoma transcript 1 (MALAT1) promotes vasculogenic mimicry and angiogenesis by increasing the expression of VE-cadherin and b-catenin on cell membranes, as well as by increasing levels of metalloproteases MMP2/9 and MT1-MMP and of phosphorylated ERK, FAK, and paxillin. These events promote gastric cancer tumor growth and metastasis [213]. LncRNA H19 is a competing endogenous RNA (ceRNA) for miR-138. Given that miR-138 targets FAK and suppresses its expression, the action of lncRNA H19 increases FAK expression and osteogenesis of bone marrow mesenchymal stem cells under tension [189]. LncRNA can also act by directly binding to proteins. Upregulation of the lncRNA CASC9 in esophageal squamous cell carcinoma (ESCC) was correlated with metastasis and poor prognosis. CASC9 upregulates the laminin subunit LAMC2 by recruiting the transcriptional coactivator CREB-binding protein (CBP) to the LAMC2 promoter. LAMC2 upregulation is associated with phosphorylation of FAK and activation of the PI3K/AKT pathway [214]. Phenotypically, CASC9 therefore produces opposing effects to those of miR-1298 (see above).

## 9. Discussion

By linking cell motility and survival, FAK and PYK2 promote the survival and invasiveness of cancer cells. In particular FAK, but also to some extent PYK2, have been recognized as anti-cancer targets. Small-molecule inhibitors that target FAK and PYK2 are currently in clinical trials. However, the efficacy and specificity of these inhibitors remain low in several cases, and their exact mechanism of action is not always well understood [10,215]. Moreover, the application of anti-cancer drugs designed to selectively inhibit single kinases can trigger large-scale signal network rewiring, leading to bypass mechanisms that render the drug ineffective [216,217]. 

Stringent endogenous control of kinase activity is essential for normal cellular function and homeostasis. Evoking naturally evolved endogenous control mechanisms for therapeutic purposes may trigger fewer escape maneuvers in diseased cells than are currently allowed by targeted kinase inhibitors. Endogenous mechanisms that (negatively) regulate the activity of FAK and PYK2 in normal or diseased cells might inspire improved or additional therapeutic interventions (Figure 2).

**Figure 4:**
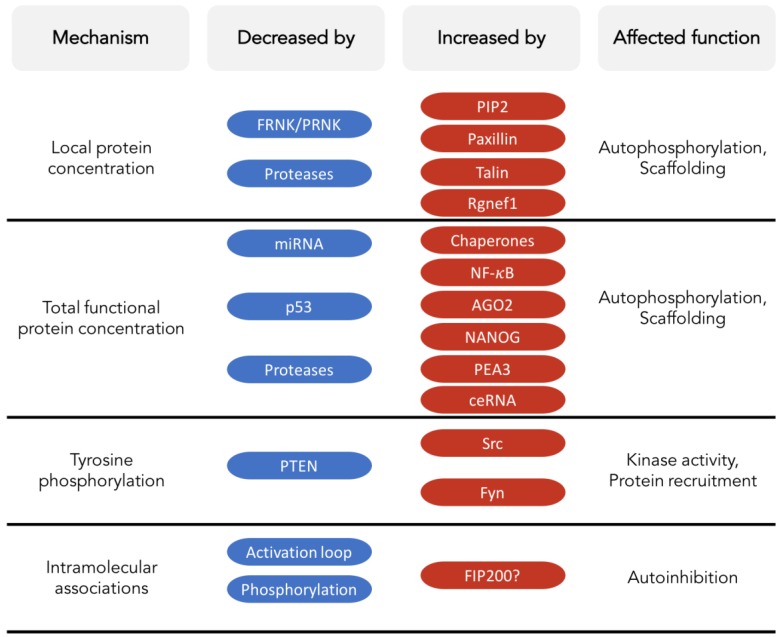
Overview of selected endogenous mechanisms that influence functions of FAK or PYK2. The ‘Mechanism’ column shows examples of features that affect FAK and PYK2 function, i.e., their local protein concentration, the total amount of correctly folded protein, the degree of tyrosine phosphorylation, and the existence and stability of intramolecular associations, in particular the FERM:kinase autoinhibitory interaction. Selected examples of factors that decrease or increase these mechanisms are shown. For FIP200, the question mark indicates that its influence on intramolecular associations is highly speculative.

FAK and PYK2 play numerous kinase-dependent and kinase-independent biological roles (Figure 4) that can vary with cellular status, cell type and subcellular location. While playing these roles, FAK and PYK2 can have opposing, redundant or synergistic effects, depending on the cell type and condition. In response, cells use different general or specifically adapted mechanisms to control FAK and/or PYK2. The general mechanisms involve chaperones, proteases, and kinases/phosphatases (such as the Src, LKB1 or PTEN). Therapeutic strategies that target these regulatory mechanisms will likely provoke pleiotropic effects, which might not always be counterproductive in diseased cells, but would generally be difficult to predict and may adversely affect healthy tissue.

Inhibitors specifically targeting the p53 or Mdm2 binding site on the FERM domain of nuclear FAK are also promising in treating cancers without deleterious mutations of p53 as indicated by an inhibitor of the putative p53 binding site on FAK FERM exhibiting tumor-suppressive effects by dampening FAK-induced p53 degradation [218]. However, the structural basis for these interactions needs to be clarified before they can be used as templates for the rational design of inhibitory small-molecule mimics.

An alternative strategy is to target the self-association mechanisms of FAK and PYK2 needed for their trans-autophosphorylation. Such allosteric protein-protein interaction inhibitors could directly manipulate the molecular control mechanisms of FAK or PYK2 without the risk of severe off-target side effects commonly associated with inhibitors of kinase domains. For FAK, such protein-protein interaction inhibitors could target the FERM or FAT domains, which are required for trans-autophosphorylation, or they could stabilize the inhibitory FERM:kinase association [31,40,46]. Indeed, compounds have been developed that target these mechanisms, including molecules that block access to Y397 or inhibit interactions of the FAT or FERM domains [10,218,219,220,221,222]. Although these compounds were found to lack efficacy and to require micromolar concentrations, they display tumor-suppressive activity in xenograft mouse models and enhance the effect of conventional chemotherapy. However, their specificity and mode of action remain poorly understood [10].

Another promising approach to utilizing endogenous mechanisms for controlling the spread and metastasis of cancer cells may be based on ncRNAs. Their mode of action through direct binding to mRNA is simple and sufficiently well understood, and detailed descriptions of their tumor-suppressive effects are rapidly accumulating. Therapeutic miRNAs or siRNAs are currently emerging as next-generation biopharmaceuticals that can rapidly move from the bench to the clinic, with about 20 molecules currently in clinical trials. Although none of these 20 ncRNAs are targeting FAK or PYK2, most are used to treat cancers, and two are specifically silencing kinases (SYK and PKN3) [223,224]. These proofs of concept of ncRNA-based therapeutics combined with the accruing evidence for the tumor-suppressive effects of miRNA-mediated inhibition of FAK and/or PYK2 suggest that the development of FAK- and/or PYK2-specific anti-cancer mi/siRNA therapies would be worthwhile. Moreover, the biological capacities of the almost 20,000 potentially functional human lncRNAs are only beginning to emerge [225]. In particular, their capacity to act as ceRNA or to directly bind to proteins (see, for example, [226]) might lead to interesting regulatory effects with therapeutic potential. Although to date no lncRNA has been discovered that directly binds to FAK or PYK2, RNA aptamers can potently and selectively inhibit kinases through positioning of an adenine nucleotide into the ATP-binding pocket [227], and more than 40 kinases have been identified as non-classical RNA binding proteins in a screen of the Arabidopsis thaliana proteome [228]. Methods for efficient and specific in vivo delivery of ncRNAs must overcome many challenges, including poor tissue penetration, rapid degradation, immunotoxicity, reliance on cellular factors required for miRNA function and possible off-target effects [229]. Nonetheless, advances in tissue delivery methods and in ncRNA bioengineering and recombinant production methods (e.g., [230]) suggest that ncRNAs are promising nature-inspired agents that can enrich our toolkit of selective therapeutic inhibitors of FAK and PYK2.

## 10. Conclusions

Despite decades of investigations, FAK and PYK2 are still revealing novel or nuanced biological functions. This versatility appears to result, at least in part, from the capacity of FAK and/or PYK2 to associate with a wide range of proteins or other molecules. These interacting molecules can activate the kinase activity, and/or utilize the scaffolding function of FAK and PYK2 in different tissues, cellular conditions or subcellular localizations, resulting in a rich arsenal of functions for both proteins. Accordingly, cells need to evoke an equally rich set of mechanisms to control the activities of FAK and PYK2. Currently these endogenous inhibitory mechanisms appear less understood than activating mechanisms. In combination with chemo-, radio- or immunotherapy FAK inhibitors are now emerging as valuable anti-tumor agents by blocking FAK and/or PYK2 from providing survival mechanisms under stress conditions, such as DNA damage or immunological stress. An increase in our molecular and mechanistic understanding of both, activating and inactivating cellular mechanisms would certainly help efforts for rationally improving the current inhibitors of FAK and/or PYK2. A thorough understanding of how these inhibitors affect FAK or PYK2 function may also allow testing their efficacy in other pathologies implicating FAK or PYK2, such as neurodegenerative diseases [231,232,233]. 

## Figures and Tables

**Table 1:** microRNAs with indirect effects on (FAK).

miRNA	Cell Type	Target Site(s)	Role	Ref.
miR-9	Ovarian serous carcinoma	TLN1, FAK, Akt	Tumor suppressor	[175]
miR-16	Glioma	p-FAK, p-Akt expression, nuclear factor-κB, Slug	Tumor suppressor	[176]
miR-17-3p	Cardiac fibroblasts	Par4, CEBPB, FAK, N-cadherin, vimentin, Oct4, Sca-1, E-cadherin	Oncogene	[177]
miR-21	Breast cancer lymph node metastasis	CDK5RAP1, CDK5 activator p39, FAK	Oncogene	[178]
miR-34a	Neuroblastoma, colorectal cancer	VEGF, FAK	Tumor suppressor	[179,180]
miR-92b	Esophageal squamous cell carcinoma	ITGAV, FAK, Rac1	Tumor suppressor	[181]
miR-124	Glioma	Capn4, -FAK, MMP2, vimentin, N-cadherin	Tumor suppressor	[182]
miR-130a	Hemangioma	TFPI2, FAK, PI3K, Rac1, mdm2	Oncogene	[183]
miR-133b	Osteosarcoma	BCL2L2, MCL-1, IGF1R, MET, FAK, Akt	Tumor suppressor	[184]
miR-134	Hepatocellular carcinoma	ITGB1, FAK and RhoA	Tumor suppressor	[185]
miR-138	Ewing’s sarcoma, head and neck squamous cell carcinoma	RhoC, FAK, Src, Erk(1/2)	Tumor suppressor	[186,187,188,189,190]
miR-141	Renal cell carcinoma	EphA2, p-FAK, p-AKT, MMP2/9	Tumor suppressor	[191]
miR-141/200c cluster	Breast cancer	VEGF-A, FAK, PI3K, Akt	Oncogene	[192]
miR-145	Glioma	CTGF, SPARC, FAK	Tumor suppressor	[193]
miR-150	Lung cancer	Src, FAK, Ras, ERK	Oncogene	[194]
miR-151-5p	Gastric cancer, hepatocellular carcinoma	FAK (host genet), RhoGDIA, Rac1, Cdc42, Rho GTPases	Oncogene	[195,196,197]
miR-187	Ovarian cancer	Dab2, E-cadherin, vimentin, FAK	Tumor suppressor	[198]
miR-199a-5p	Breast cancer	Ets-1, FAK/Src/Akt/mTOR	Tumor suppressor	[199]
miR-202	Esophageal squamous cell carcinoma	LAMA1, FAK-PI3K-Akt	Tumor suppressor	[200]
miR-221 and miR-26b	Mesenchymal stem cells	PTEN, FAK, PI3K, Akt		[201]
miR-296-3p	Lung Adenocarcinoma	PRKCA, FAK-Ras-c-Myc	Tumor suppressor	[202]
miR-375	Mesenchymal stem cells	FAK, paxillin, PDK1, Akt		[203]
miR-383	Glioma	VEGF/VEGFR2, FAK, Src	Tumor suppressor	[204]
miR-425-5p	Hepatocellular carcinoma	SCAI, integrin β1-Fak/Src-RhoA/CDC42, PTEN-AKT, TIMP2-MMP2/MMP9	Oncogene	[205]
miR-491-5p	Oral squamous cell carcinoma	GIT1, paxillin, FAK, EGF/EGFR- ERK1/2, MMP2/9	Tumor suppressor	[206]
miR-542-3p	Colon cancer	ILK, FAK/c-Src	Tumor suppressor	[207]
miR-647	Gastric cancer	ANK2, FAK, MMP2, MMP12, CD44, SNAIL1,	Tumor suppressor	[208,209]
miR-708	Metastatic breast cancer	Neuronatin, ERK, FAK	Tumor suppressor	[210]
